# Genomic Scars Generated by Polymerase Theta Reveal the Versatile Mechanism of Alternative End-Joining

**DOI:** 10.1371/journal.pgen.1006368

**Published:** 2016-10-18

**Authors:** Robin van Schendel, Jane van Heteren, Richard Welten, Marcel Tijsterman

**Affiliations:** Department of Human Genetics, Leiden University Medical Center, Leiden, The Netherlands; The University of Texas MD Anderson Cancer Center, UNITED STATES

## Abstract

For more than half a century, genotoxic agents have been used to induce mutations in the genome of model organisms to establish genotype-phenotype relationships. While inaccurate replication across damaged bases can explain the formation of single nucleotide variants, it remained unknown how DNA damage induces more severe genomic alterations. Here, we demonstrate for two of the most widely used mutagens, *i*.*e*. ethyl methanesulfonate (EMS) and photo-activated trimethylpsoralen (UV/TMP), that deletion mutagenesis is the result of polymerase Theta (POLQ)-mediated end joining (TMEJ) of double strand breaks (DSBs). This discovery allowed us to survey many thousands of available *C*. *elegans* deletion alleles to address the biology of this alternative end-joining repair mechanism. Analysis of ~7,000 deletion breakpoints and their cognate junctions reveals a distinct order of events. We found that nascent strands blocked at sites of DNA damage can engage in one or more cycles of primer extension using a more downstream located break end as a template. Resolution is accomplished when 3’ overhangs have matching ends. Our study provides a step-wise and versatile model for the *in vivo* mechanism of POLQ action, which explains the molecular nature of mutagen-induced deletion alleles.

## Author Summary

DNA damage poses a threat to cell survival as it impedes accurate and efficient copying of DNA that precedes mitotic division. If left unrepaired, DNA damage can give rise to DNA double-strand breaks (DSBs), which is considered to be one of the most dangerous types of genomic insult. Repairing DSBs is vital to preserve genomic integrity and to promote cellular survival. Here, we demonstrate in *C*. *elegans* that exposure to mutagenic agents leads to replication-associated DSBs that require polymerase Theta (POLQ)-mediated end joining for their repair. We provide a mechanistic basis for understanding how mutagens that interfere with DNA replication induce the type of deletion mutations that have been generated in a great variety of organisms in order to establish genetic null alleles. Bio-informatical data-mining of many thousands of deletion alleles subsequently has allowed us to establish DNA repair pathway-specific signatures and to uncover a surprisingly simple and elegant solution of how cells can repair a broken chromosome, while keeping the loss of genetic information to a minimum.

## Introduction

DNA mutations fuel evolution of organisms giving rise to speciation, and of cells within an organisms giving rise to cancer. Two replication-associated mechanisms are responsible for most if not all single nucleotide variants (SNVs) as well as small insertions/deletions (indels) at repetitive sequences: i) copying errors made by the replicative polymerases delta and epsilon, which are mostly undone by DNA mismatch repair, and ii) replication of damaged DNA by specialized so-called translesion synthesis (TLS) polymerases. TLS polymerases, in contrast to the replicative polymerases, have the ability to extend nascent DNA strands across non- or poorly coding damaged bases, often leading to mutation. It is, however, less well understood which mechanisms are responsible for other types of genomic alterations, such as deletions that are larger than a few bases.

A recent study that involved whole genome analysis of *C*. *elegans* animals that were propagated for many generations revealed that vast majority of accumulating deletions larger than 1 bp required the activity of the A-family polymerase Theta (POLQ). Upon unperturbed growth, wild-type *C*. *elegans* genomes accumulate SNVs as well as deletions but the latter class was strikingly absent in strains that were defective for POLQ [1]. Instead, much more dramatic chromosomal rearrangements were noticed indicating that POLQ action protects the genome against deterioration but at the cost of a small genomic scar. A similar profile of mutagenesis was observed resulting from DNA double-strand break repair, which hinted towards DSBs as being a very prominent source of genome diversification during evolution, and towards error-prone DSB repair as the mechanism responsible for this type of genome alterations [1].

The first demonstration of POLQ acting on DSBs was made in *Drosophila*: *in vivo* processing of artificially-induced DSBs in POLQ-mutant flies deviated from that in wild-type flies [2]. POLQ deficiency did not increase sensitivity to ionizing radiation, yet it did greatly exacerbate hypersensitivity in flies impaired in homologous recombination. Apparently, a POLQ-dependent DSB-repair pathway can act as a backup in HR-compromised circumstances. Indeed, recent work on human POLQ revealed a strong synergistic relationship between the HR pathway and POLQ-mediated DSB repair [3,4]. The synthetic lethal nature of this genetic interaction may be of great clinical importance as it identifies POLQ as a druggable target for tumours carrying mutations in HR genes. Another indication that POLQ repairs DSBs in contexts where HR is compromised came from genetic studies performed in *C*. *elegans*. Here it was shown that POLQ-mediated repair is the only pathway (also in HR-proficient conditions) capable of repairing replication-associated DSBs that are induced when persistent DNA damage or stable secondary structures cause a permanent block to DNA replication [5,6]. It was subsequently shown that these DSBs result from inheritable ssDNA gaps opposite to the strand containing the damage, which could thus not serve as a template for HR [7].

Extensive analyses of repair products in both flies and worms provided a clear signature of POLQ-mediated DSB repair with two prominent features: i) the notion of microhomology at the repair junctions, a feature previously ascribed to non-canonical end-joining also called alternative end-joining [8,9], and ii) the occasional presence of so-called template inserts: deletions that contain, at the deletion junction, the inclusion of a DNA insert (hereafter called delins). These inserts are of variable length but their origin can be mapped to DNA regions that lie in very close proximity to the DSBs ends that produced the delins. Similar hallmarks can be found for POLQ-mediated DSB repair in human and mouse cells [4,10]. A recent *in vitro* study provided a molecular explanation for the prominent presence of microhomology at the DSB repair junctions: repair reactions with purified protein showed that two base pairs of complementarity is enough for human POLQ to pair and extend 3’ overhangs of partially double-stranded oligonucleotides [11].

Although it is now becoming increasingly clear that POLQ plays an evolutionarily conserved role in DSB repair, how POLQ acts *in vivo* to explain all the observed consequences remains to be elucidated. Over the last four decades, the *C*. *elegans* community has used EMS and UV/TMP to generate many thousands of deletion alleles, but the underlying mechanism has remained unknown. Here, we demonstrate that mutagen-induced replication breaks in *C*. *elegans* germ cells are exclusively repaired by POLQ. This publically available allele collection, reflecting ~7,000 *in vivo* POLQ-mediated end joining reactions, allows us to analyse and describe the POLQ-mediated repair mechanism in great detail.

## Results

### POLQ-deficient animals are hypersensitive to EMS and UV/TMP

**Fig 1:**
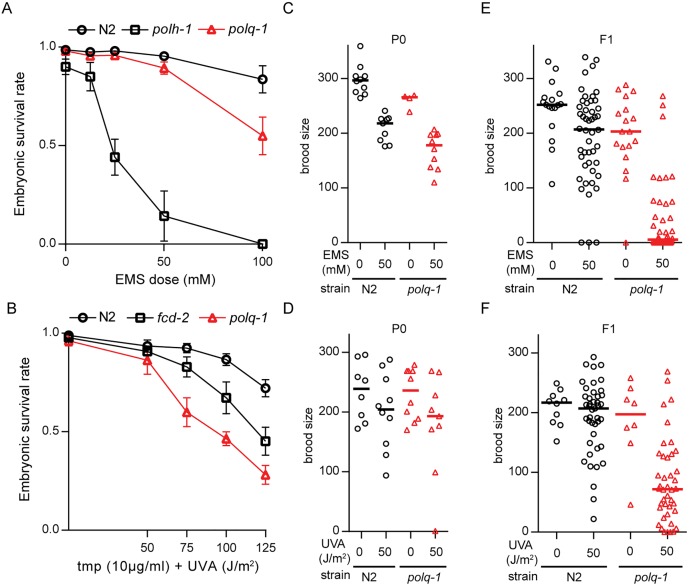
POLQ-deficient animals are hypersensitive to EMS and UV/TMP. **A.** Sensitivity to EMS exposure. **B**. Sensitivity to UV/TMP treatment. L4 animals of the indicated genotype were exposed to DNA damaging treatments and survival was quantified by counting dead embryos versus living progeny in the next generation. **C-D**. The total brood (eggs + larvae) was determined for P0 animals of the indicated genotype that were mock treated or treated with EMS (C) or UV/TMP (D). Lines represent the median for each dataset. **E-F**. The total brood was determined for F1 animals that originated from P0 animals that were either mock treated or treated with EMS (E) or UV/TMP (F). Lines represent the median for each dataset.

To investigate whether POLQ plays a general role in the processing of mutagen-induced DNA damage, we assayed embryonic survival in animals that were exposed to two of the most widely used mutagens in *C*. *elegans*: EMS, which causes alkylating damage, and TMP, which, upon exposure to UVA light, results in monoadducts and crosslinks. We found *polq-1*-deficient animals to produce more unviable embryos than wild-type animals when exposed to EMS (Fig 1A and S1 Fig), but not to the extent observed in animals that are defective for polymerase eta (*polh-1*), a translesion synthesis (TLS) polymerase that is involved in replicative bypass of DNA damage [12]. A similar mild hypersensitivity was observed when *polq-1*-mutant animals were incubated with TMP and subsequently exposed to UVA (Fig 1B and S1 Fig), in agreement with previously published work [13]. In addition to monitoring the survival of embryos, we monitored their ability to produce functional gametes. Complete or partial sterility of daughters from exposed mothers is another phenotype that is related to genotoxic stress, likely because germ cells, or their progenitors, are more susceptible to DNA damage-induced arrest, apoptosis, and mitotic catastrophe [14]. Indeed, at EMS or UV/TMP doses where the brood size of exposed mothers were only moderately affected in both wild-type and *polq-1*-mutant animals (Fig 1C and 1D) dramatic sterility was observed in *polq-1* but not in wild-type progeny animals (Fig 1E and 1F): 99% versus 16% median reduction, in brood for EMS-treated animals, and 65% versus 5% for UV/TMP-treated animals. These data establish a prominent role for POLQ in protecting germ cells against EMS and UV/TMP-induced toxicity.

### EMS and UV/TMP-induced deletions are dependent on POLQ

**Fig 2:**
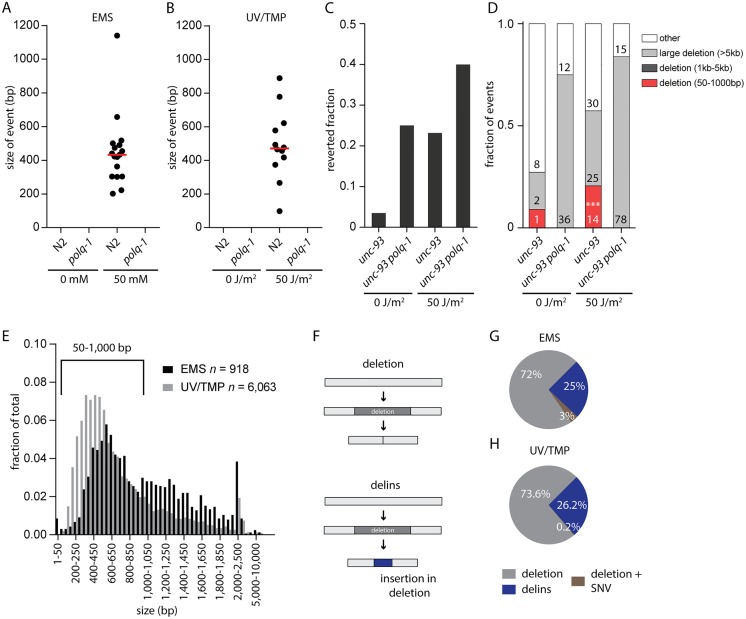
EMS and UV/TMP-induced deletion alleles are dependent on POLQ. **A-B.** Size distribution for all confirmed deletion events found in EMS (A) or UV/TMP (B) mutagenized libraries. Red bars represent the median deletion size. **C**. Fraction of populations that contained *unc-93(e1500)* revertant animals. At least 250 populations were assayed per experimental condition. **D.** Distribution of *unc-93* reversion-footprints for the indicated genotype and experimental condition. The class of 50-1000bp was found to be statistically different between treated *unc-93* and *unc-93 polq-1* animals. The category ‘other’ includes wild-type sized PCR products, which based on previous experiments mostly reflect base substitutions. (p<0.001, Fisher’s exact test, indicated by ***) **E**. Size distribution of EMS- and UV/TMP-induced deletions generated by the *C*. *elegans* community. Only the deletions 50–1,000 bp (918 and 6,063 for EMS and UV/TMP-induced deletions, respectively) were used in subsequent analyses. **F**. Graphic representation of the two different types of deletions. The upper panel illustrates a simple deletion, in which only sequence is lost; the bottom panel reflects a delins, in which loss of sequence is accompanied with the insertion of *de novo* sequence. **G-H**. Pie chart representation of the fraction of deletions and delins that were isolated from EMS (G) and UV/TMP (H) mutagenized libraries. Deletions + SNV represent cases where a SNV is found in close proximity to a deletion.

EMS and UV/TMP are widely used mutagens in *C*. *elegans* to create loss-of-function alleles [15]. Given the sensitivity of *polq-1* animals towards these agents we wanted to investigate whether POLQ functionality is relevant for generating these alleles. EMS predominantly alkylates guanine which can be bypassed, leading predominantly to GC>AT transitions [15–17]. Deletions also result from EMS treatment through yet unknown biology [17]. UV/TMP treatment results in a different spectrum of mutations: for this mutagen, deletions dominate base pair substitutions [17,18], but also here, the underlying mechanism of deletion formation is unknown. To address the candidate role of POLQ in producing deletion alleles, we created libraries of mutagenized wild-type and *polq-1*-mutant animals and screened them for deletions. We used standard protocols that were previously used by numerous laboratories and consortia leading to the ~10,000 *C*. *elegans* deletion alleles that are currently available [19–21]. The general concept of these protocols is to find by PCR a smaller than wild-type product for a target of interest in pooled broods of mutagenized animals; then use a sib-selection strategy to isolate the mutant allele (S2 Fig and Methods section). Because the progeny of mutagenized *polq-1*-animals have a reduced brood size (Fig 1E and 1F), we screened the F1 generation, and not the F2, which allowed us to inspect the same number of animals for *polq-1*-mutant and wild-type genotypes. We screened the libraries for deletions using eight different amplicons, all ~1 kb in size. Positive pools were chased by PCR of less-complex pools and individual library addresses (in duplicate) to exclude false positives (See Methods for details). This strategy proved to be robust and specific as deletion alleles were readily detected in wild-type animals exposed to either EMS or UV/TMP, but not in mock-treated animals (Fig 2A and 2B and S2B and S2C Fig). In contrast, we did not find a single deletion allele in libraries of either EMS- or UV/TMP-mutagenized *polq-1* animals (Fig 2A and 2B). From this data we conclude that EMS- and UV/TMP-induced deletion mutagenesis, in the size range of 50 bp up to ~1 kb, requires functional POLQ.

To further validate this conclusion we investigated UV/TMP-induced mutagenesis in a more unbiased fashion by catching loss-of-function mutations in an endogenous genomic target, *unc-93*. A dominant mutation in the transmembrane protein UNC-93, *unc-93(e1500)*, causes worms to move uncoordinatedly. Loss of UNC-93 expression, or of one of its cofactors SUP-9 and SUP-10 results in a reversion to wild-type movement, which provides an easy phenotypic manner to monitor loss of function mutagenesis. We exposed POLQ-proficient and -deficient animals, carrying the *unc-93(e1500)* allele to TMP with or without UVA irradiation to introduce crosslinks. Wild-type-moving animals were isolated from the brood of exposed animals and subsequently inspected for deletions in *unc-93*, *sup-9* and *sup-10*. The mutants that did not, by DNA gel electrophoresis, reveal a deletion in any of the three genes are likely the result of single nucleotide variations (SNVs) and were not further analysed. In treated wild-type animals, we observed an increase in two distinct categories of deletions (Fig 2C and 2D): one class, comprising of small, 50 bp to 1 kb, deletions with median size of ~100 bp (S2D Fig), and another class in which deletions are substantially larger, being >5 kb in size (Fig 2D). No deletions were found in the size range 1–5 kb. UV/TMP-treated *polq-1*-deficient animals were, however, devoid of small deletions, while the ratio of very large deletions further increased (Fig 2C and 2D). Based on these data and the PCR-based screenings of UV/TMP-treated mutant libraries, we conclude that the vast majority (if not all) of small deletions in the range of 50 bp up to at least 1 kb are the result of POLQ action. In its absence large deletions manifest, which, in agreement with our previous work, argue that POLQ prevents large genomic alterations at replication blocking DNA lesions at the expense of relatively small deletions [1,5,6].

### Replication approaches to one nucleotide from the damage

Above, we demonstrate that deletion alleles isolated from libraries of EMS- and UV/TMP-treated populations are the result of POLQ action. This notion allows us to systematically analyse a uniquely rich collection of ~2,000 EMS- and ~8,000 UV/TMP-induced deletion alleles that were generated by the *C*. *elegans* community to elucidate the *in vivo* mechanism of POLQ action. Fig 2E displays the sizes for all ~10,000 alleles, for which the sequence information was retrieved from WormBase [22]. The majority of alleles are between 50 bp and 1kb and can be categorized into two groups: i) simple deletions, which make up the majority of events (~70–75%) in both the EMS and in the UV/TMP dataset, and ii) deletions that are accompanied by an insertion of a small segment (median: 5 bp for both sets) of novel DNA; we refer to this class (~25–30%) of alleles as delins (Fig 2F–2H). We set out to characterize the ~5,000 deletions and ~1,800 delins, filtered to size (50–1,000 bp), into great detail.

**Fig 3:**
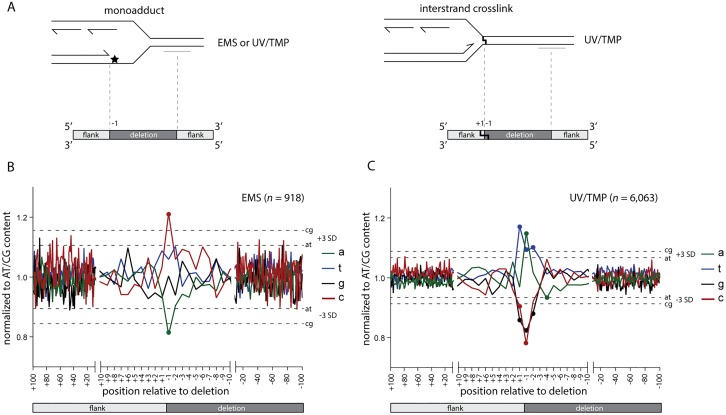
Replication approaches to one nucleotide from the damage. **A.** Schematic illustration of the concept that one junction of DNA-damage-induced deletions is defined by the nascent strand blocked at sites of DNA damage. In this hypothesis, the replication-blocking lesion may dictate position -1, being the outermost nucleotide of the lost sequence. **B-C**. The base composition of all breakpoints, normalized to the relative AT/CG content around the breakpoints (from +100 to -100). Position +100 to +1 reflects the sequence that is retained in the deletion alleles; position -1 to -100 reflects the sequence that is lost. Dashed lines represent three times the SD. Data points outside these boundaries are marked with a dot.

First, we investigated the base composition of deletion junctions to further examine an earlier reported relationship in POLQ-mediated mutagenesis between the position of a deletion breakpoint and the position of a replicating blocking lesion: we previously found for deletions resulting from replication blocking G-quadruplexes that one of the breakpoints maps close to the replication impediment [6]. This led to a model where deletions result from processing the 3’ hydroxyl ends of blocked nascent strands. DNA lesions induced by EMS and UV/TMP also have the potential to block replication, and we thus questioned whether cognate deletions close to their breakpoints carry the signature of EMS- or UV/TMP-inflicted base damage. More precisely, if one of both breakpoints results from processing a stable but reactive nascent strand that was extended up to the damaged base, then the first nucleotide immediately downstream of the breakpoint (the -1 position) might reveal the nature of the replication impediment (see Fig 3A for a graphical illustration of this concept). Indeed, we found a clear non-random base composition at position -1: for EMS we found an overrepresentation of cytosine (Fig 3B and S3 Fig), which perfectly fits the damage spectrum of EMS predominantly ethylating guanines [16,17]. Blocked DNA synthesis, incapable of extending across a damaged guanine, would result in a 3’ hydroxyl end immediately upstream of a cytosine. Also for deletions induced by UV/TMP we found at the -1 position a clear mutagen-specific overrepresentation of a particular base, in this case an adenine (Fig 3C), which reflect TMPs reactivity towards thymines [23]. Strikingly, and in contrast to the EMS spectrum, we here also observed a non-random distribution at the +1 position, being a thymine. This outcome suggests that UV/TMP-induced deletions are preferentially induced at sites where replication is blocked by a thymine that is preceded by an adenine, a conclusion that is further supported by probing the datasets with pairs of nucleotides (S3 Fig). This prevalent signature is in perfect agreement with the preference of psoralens to intercalate into and react with 5’TA in duplexed DNA [24,25]. Without further genetic dissection, however, it is impossible to discriminate between interstrand crosslinks at 5’TA sites or monoadducts (or DNA-protein complexes) formed at sites of preferred intercalation, being responsible for POLQ-dependent deletion formation. Irrespective which lesion, our data indicates that replication can proceed right up to the base that is damaged by the psoralen moiety.

Our analysis of ~7,000 mutagen-induced deletion alleles reveals a clear lesion-specific signature in POLQ-mediated deletion formation. Importantly, a single replication fork block triggers such a deletion, as we observed a damage signature at only one of both breakpoints (S4 Fig). The position of the damage with respect to the deletion junction supports a mechanistic model where the nascent strand blocked at the site of base damage is not subjected to extensive trimming but instead is reactive towards a POLQ-mediated end-joining reaction that has small sized deletions as an end-product. The putative mechanism responsible for generating the other reactive end at a 50–1,000 bp distance will be discussed later, but we will provide evidence that, with respect to reactivity, it is indistinguishable from the blocked nascent strand.

### Single nucleotide priming is sufficient to initiate repair by POLQ

**Fig 4:**
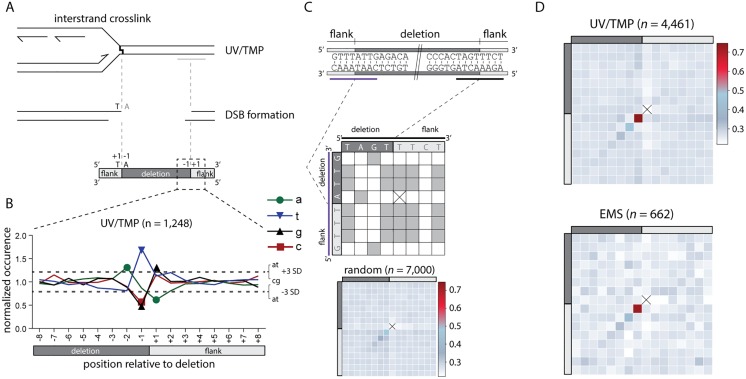
POLQ-mediated repair is characterized by single nucleotide homology. **A**. Schematic illustration of a replication fork blocked at an UV/TMP-induced crosslink that subsequently leads to a DSB, which is repaired by POLQ leading to a deletion of the intervening sequence. One reactive end of the DSB is determined by the nascent strand blocked by an UV/TMP-induced crosslink that predominantly links thymines in opposite strands when in a 5’TA configuration. **B**. Deletion alleles that contain a 5’TA at the (+1, -1) position of one of their breakpoints are analysed (*n* = 1,248) for the base composition at the opposite breakpoint. Dashed lines represent three times the SD, which is determined by the base composition of the region between -100 and +100. **C**. Schematic illustration of how microhomology between breakpoints is determined in an unbiased manner. For each allele a table is constructed that allows for the scoring of homology between both breakpoints that give rise to a deletion. Each position of the upstream breakpoint (purple) is compared to each position of the downstream breakpoint (black). Identical nucleotides score 1, non-identical score 0. Subsequently, a heat map is constructed by summing all scores for all events at each position divided by the number of events. For reference purposes, a heat map was constructed for 7,000 deletions randomly created *in silico* throughout the genome. Of note, all alleles are annotated in keeping with maximal 5’ conservation, which here dictates that the base at the -1 position at the 5’ side is never identical to the +1 position at the 3’ side: in such a case, that base will shift to the +1 position at the 5’ side. As a consequence of this rule, the position marked by a cross will have no microhomology score, while the +1,-1 position is slightly elevated. The extent of this methodological skewing can be noticed in the analysis of the random set of deletions. **D**. Heat maps for UV/TMP- and EMS-induced deletions. Heat map contains 16 bases overlapping each breakpoint; 8 bases immediately flanking the deletion (light grey) and 8 bases immediately inside the deletion (dark grey).

We reveal above that the terminal nucleotide of the nascent strand, blocked at the site of base damage, is retained in the repair product, it is the base immediately flanking the deletion, but does it also guide repair? To address this question we compiled all simple deletions from the UV/TMP dataset that had the signature T_+1_,A_-1_ composition at one of both breakpoints, because only for this subclass (*n* = 1,248) the identity of the terminal nucleotide of the nascent strand is known, i.e. a thymine. We then tested the following prediction: if this 3’ thymine is guiding repair of the break, by providing a minimal primer for POLQ, a thymine should be overrepresented at the -1 position of the opposite flank (Fig 4A for a graphical illustration). This is indeed what we found: Fig 4B shows that the composition of the donor sequence opposite to the blocked nascent strand is completely random apart from position -1, which is dominated by a thymine. A similar conclusion results if we use an approach that is blind to the replication-obstructing base and does not restrict the analysis to a single nucleotide. For each of the ~5,000 alleles we established the degree of homology between both breakpoints by scoring the degree of sequence identity in a 16-nt window, encompassing the 8 outermost nucleotide of the flanking sequence and the 8 nucleotides of the adjacent but deleted sequence (see Fig 4C for a schematic illustration of the approach). These plots were subsequently compiled to generate heat maps for the different category of alleles. In both the UV/TMP-induced (*n* = 4,461) and the EMS-induced deletions (*n* = 662) crosstalk between both breakpoints is observed, but only for the nucleotide at the -1 position of the deletion and the +1 position of the opposing flank (Fig 4D). This outcome lends further support to the hypothesis that the terminal base of one end, upon minimal pairing with the opposing template, is guiding POLQ-mediated repair.

### Templated inserts and simple deletions have a common origin

**Fig 5:**
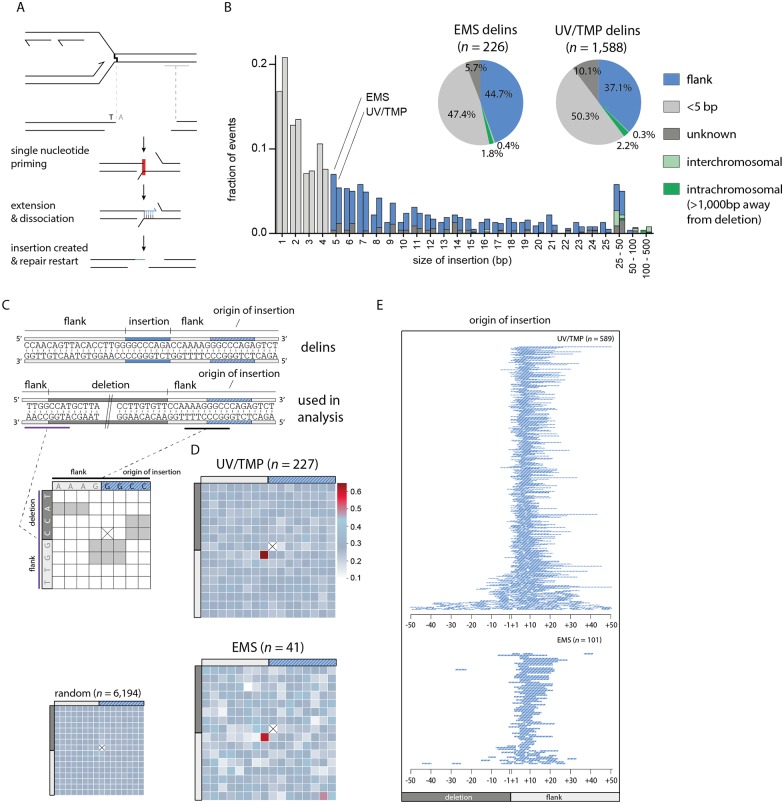
Hallmarks and genesis of delins. **A**. Schematic illustration of the concept that templated insertions are generated by POLQ-mediated extension of one reactive 3’ end (*e*.*g*. the nascent strand blocked at sites of base damage) using the other end as a template: single nucleotide priming and disrupted extension can lead to delins formation. **B.** Size distribution of insertions found in EMS- and UV/TMP-derived delins. For 47–50% of delins the insert size is too small (<5 bp) to uniquely identify their origin. 37–44% of delins can be mapped to within 20 bp flanking the breakpoint. Another 2–3% of delins are copied from inter- or intrachromosomal (>1000 bp away from deletion) locations. For 6–10% of delins no apparent source could be identified. **C**. Schematic illustration for how microhomology is determined between the sequence that was used as a template for the generation of an insertion (the template) and the opposite breakpoint (the primer). A typical delins is portrayed at the sequence level as an example in which both the insertion (in blue) as its identified origin (in striped blue) is indicated. Underneath is another representation of the same delins, now containing the deleted sequence. This configuration is used in the subsequent analysis, where for each delins a table is constructed in which the bases overlapping with the 5’ side of the insertion origin (black) are compared to the bases that are overlapping the opposite breakpoint (purple). Identical nucleotides score 1, non-identical score 0. Subsequently, a heat map is constructed by summing all scores for all events divided by the number of events at each position. For reference purposes, a heat map was constructed for ~6,000 delins with perfect templated flank insertion randomly created *in silico* throughout the genome. Of note, at one position such a comparison cannot be done because the start and end nucleotide of an insertion is never identical to the deleted part of a delins and are thus always 0 (crossed out). As a result some other positions become slightly overrepresented as can be appreciated from the *in silico* generated delins. **D**. Heat map for UV/TMP- and EMS-induced delins for which the origin of the inserts are mapped. **E**. Visual representation of the origins of flank insertions for UV/TMP- and EMS-induced delins. A single line represents one mapped flank insertion and is drawn relative to its cognate breakpoint with ‘-’ for deleted and ‘+’ for retained sequences.

Once priming has been established and extension has commenced there are two possible fates: i) continuation and further processing; in which case the outcome will be a deletion with single nucleotide identity at the junction, or ii) discontinuation. If, in the latter case, the extended end serves as a new nucleation site for yet another round of POLQ-mediated repair, templated inserts will result (Fig 5A). If so, delins are suspected to have some features identical to those described above for simple deletions. To address this, and to further dissect the *in vivo* mechanism of POLQ-dependent mutagenesis, we characterized the ~25–30% of mutagen-induced deletion alleles that are accompanied by small insertions in great detail. First we placed them, based on their size and suspected origin, in different categories (Fig 5B): ~47–50% are so small (<5 bp) that their origin is untraceable, and another 5–10% are larger in size but their sequence does not provide enough certainty as to their origin. However, ~40–45% of delins (~700) have inserts with sufficient sequence information to reveal their source: apart from a small percentage (~3%) that comprise of sequences mapping to distant sites at the same chromosome or to other chromosomes (S5 Fig), the majority (~37–44%) maps very close to the deletion. These insertions are either completely or partially identical to parts of the flanking sequences and have been designated ‘templated inserts’ because of a presumed role for the flanking DNA to serve as a template for a repair reaction. Because the majority of templated inserts map a few bases away from the deletion junction (the template is located within the flank) a number of parameters can be investigated centred around the questions: i) what defines the start of POLQ-mediated DNA synthesis, ii) what defines the end, and iii) how accurate is it?

With respect to the start, we focused on templated inserts that are 100% identical to sequences in their flanks to avoid possible ambiguity in interpretation. For both UV/TMP and EMS-induced alleles (n = 227 and 41, respectively) we found that templated inserts, similar to simple deletions, are primed by a single base pair. This priming becomes apparent when the base composition of one breakpoint is plotted to the base pairs that are neighbouring the sequence that served as a template for extension (Fig 5C and 5D). Overrepresentation of sequence identity is confined to one position, the +1 base of one breakpoint (the reactive end) and the base flanking the origin of the insert in the opposite breakpoint (the template), providing further confirmation that a single base pair is sufficient to drive POLQ-mediated repair. We found that ~85% of inserts originate from priming within 10 base pairs of the breakpoints (Fig 5E), which could point to homology search close to the end of the available sequence.

### Templated inserts result from template switching and reiterated priming

The observed similarities in the initiation steps of deletions that are simple and those that include a templated insert means that the difference between both outcomes is the consequence of a downstream step, for instance, discontinuity of POLQ action. The determinants influencing discontinuity in the repair reaction are currently unknown but it is a remarkable frequent event as ~25% of all alleles have insertions. From plotting the size of all inserts (Fig 5B), we infer that templated inserts do not have a minimal length: although it is impossible to reliably map inserts of only one or a few bases to the flanking sequences, we observe that the percentage of inserts that can be mapped is constant, yet high, over the complete range of small insert size. This notion argues that also the very small, unmappable, insertions are flank-derived. Fig 5B also shows that while template inserts are overall rather small (<25 bp), they do not have a preferred size. Instead, a gradual decline in length is observed which may suggest that comprehensive extension prevents discontinuity. Still, we also found inserts where stretches of more than 20 consecutive bases have been templated, indicating that substantial base pairing can still be disrupted before the two opposite ends are irreversibly connected. Whether POLQ dissociates from the template in this process or whether POLQ facilitates template switching is an interesting question as the latter option could serve to broaden the resolving potential of POLQ-mediated repair. Some delins have complex combinatorial inserts with two or more mostly overlapping templated inserts, arguing for reiterative steps of priming, extension and dissociation. In most of these cases (16 out of 17) only one flank provided the template, which hints towards directionality in POLQ-mediated resolution.

**Fig 6:**
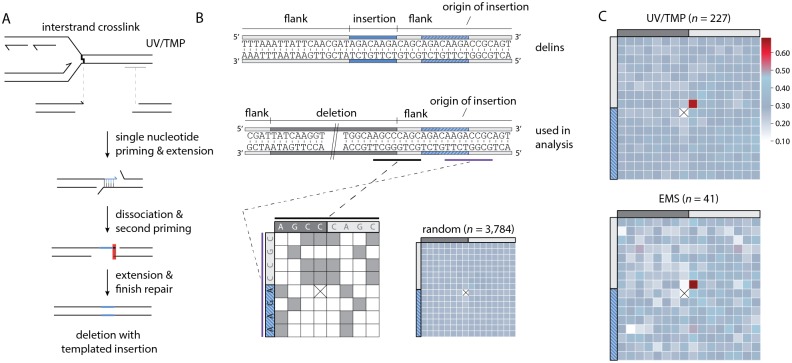
Primer-template switching results in delins formation. **A**. Schematic illustration of how primer template switching followed by POLQ-mediated extension and resolution results in a templated insertion. The requirement of single-nucleotide homology in POLQ-mediated end joining predicts that the nucleotide directly 3’ of the templated insertion (blue line) is typically identical to the outermost nucleotide of the ‘acceptor’ breakpoint. This prediction is highlighted by the red box. **B**. As in Fig 5C, but here for the end of the origin of templated insert and the adjacent deletion junction. As an example a typical delins is portrayed at the sequence level in which both the insertion (in blue) as its identified origin (in striped blue) is indicated. Underneath is another representation of the same delins, now containing the deleted sequence. This configuration is used in the subsequent analysis. Of note, at one position such a comparison cannot be done because the start and end nucleotide of an insertion is never identical to the deleted part of a delins and are thus always 0 (crossed out). As a result some other positions become slightly overrepresented as can be appreciated from the *in silico* generated delins. **C**. Heat map for UV/TMP and EMS-induced delins where the insertion origin could be faithfully traced back to the immediate flank.

To complete repair of aborted reactions, it seems plausible that another round of priming and extension is required, analogous to the biology leading to simple deletions, only in this case, one end has been extended using the other end as a template. To test this hypothesis, we again created heat maps, but here compared the terminal bases of the origin of the template inserts as well as their flanking bases (as this constitutes the new reactive end), to the border of the same flank, which in this scenario is considered the opposing end (Fig 6A). We indeed found support for a single base pair priming reaction as also here a clear overrepresentation of single nucleotide identity is observed (Fig 6B and 6C). Our combined analysis thus supports a model, where simple deletions and template inserts result from the same chemistry, displaying the same features, the only difference being an aborted POLQ-mediated extension of a single base paired-primed intermediate.

Probing the entire collection of ~10,000 EMS- and UV/TMP-induced *C*. *elegans* deletion alleles for single nucleotide identity at break junctions and the presence of template inserts suggest that POLQ-mediated end joining is responsible for the majority of deletions in a 50–3,000bp range (S6 Fig).

### POLQ activity is error prone

**Fig 7:**
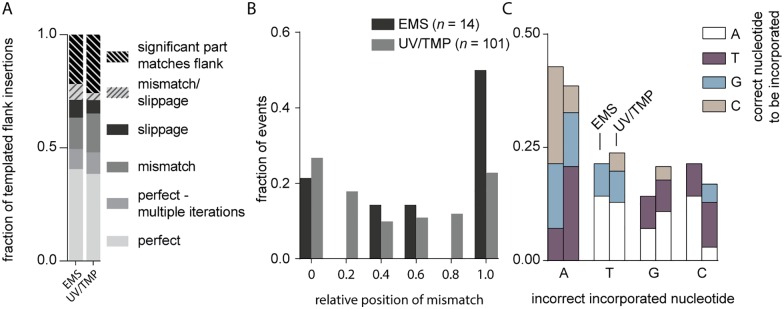
POLQ activity is error prone. **A**. The fraction of templated flank insertions derived from a single origin is greatly increased when we allow a SNV or a slippage-event in a microsatellite (≥4 bp). **B**. The relative position of mismatches in delins is plotted for each mutagen relative to the insertion. **C.** Fraction of incorrect incorporated nucleotides in EMS and UV/TMP deletions, grouped by nucleotide misincorporation.

At present it is unknown what underlies the discontinuity in POLQ-mediated repair that leads to delins instead of simple deletions. One possibility is polymerase errors. POLQ is a relatively error-prone polymerase generating single base errors at rates 10- to more than 100-fold higher than other polymerase A family members [26]. Mismatches resulting from wrongly incorporated nucleotides may reduce POLQ’s processivity and promote dissociation and/or template switching. One observation provides strong support for such a scenario: the frequency of errors observed in templated inserts is extremely high as compared to mutations in the flanks of the simple deletions, while for both repair products the flank has served as a template for POLQ action. Although ~30% of all templated inserts are perfect, in the sense that they do not show mismatches, another 15% can be matched to the flank through a single run of consecutive bases if one mismatch or one slippage event is allowed (Fig 7A). It can thus be argued that at least 1 in 3 templated inserts suffers from a mutation which translates to an error rate of ~1 in 30 base pairs during templated extension (average insert size = ~10bp). In sharp contrast, we found only few mutations in the flanks of ~4,500 UV/TMP- induced simple deletions. Assuming that here POLQ is required to extend the reactive end with at least 10 bp, we calculate an error rate of <1 in 3,000 bp for simple deletions. To explain the >100 fold higher mutation frequency in extension leading to templated inserts, we propose that POLQ errors in fact provoke template switching, thus are causal to the formation of delins. A supporting observation is that mismatches are more frequently found closer to where the reaction is abrogated (Fig 7B).

POLQ replication errors could result from replicating non-damaged or damaged DNA. The *in vitro* demonstrated bypass activity of POLQ may help to extend past base damage or abasic sites. We mostly found incorrect incorporation of adenines opposite to any nucleotide other than a thymine (Fig 7C), making up for half of all mismatches, which fits with the preferential incorporation of adenine that has been observed for POLQ *in vitro* [27].

### Mutagen-induced deletions are the product of DSB repair

**Fig 8:**
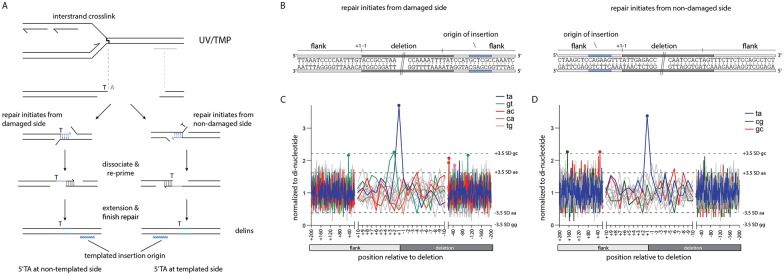
Mutagen-induced deletions are the product of DSB repair. **A**. Schematic illustration of a replication-blocking lesion that is converted to a DSB and finally results in a templated flank insertion. The 5’TA causing the deletion defines one end of the break, while the composition of the other end is unknown. By using the 5’TA together with the side of origin of templated insertions we can determine the reactivity of both 3’ break ends: if the 5’TA is on the opposite side of the templated insertion origin, repair initiated from the damaged side. On the other hand if both are on the same side then repair is initiated from the non-damaged side. **B**. Examples of two delins, portrayed at the sequence level, where either the 5’ side (left drawing) or the 3’ side (right drawing) potentially served as a primer to initiate repair. **C**. Analysis that probes the (+1,-1) junction of the side opposite to the flank containing the insertion origin. Dashes lines represent 3.5 times the SD. Only the largest and smallest variations for individual dinucleotides are shown. Only dinucleotide sets containing at least one position (marked by dots) that is >3.5 times the SD are shown in color. **D**. As in C, but in this case the (+1,-1) junction of the side that contains the insertion origin is analysed.

Finally, using this unique dataset of ~7,000 *in vivo* POLQ reactions we re-evaluated the assumption that POLQ acts to protect against mutagen-induced damage by acting on replication-associated DSBs. Despite having demonstrated that POLQ-mediated end joining is a stand-alone DSB-repair pathway that is able to process bona fide DSBs [1], it remained difficult to formally prove that a DSB is an intermediate in a repair reaction that produces simple deletions and templated inserts that were previously also found to accumulate in mutants defective for TLS polymerases. Through combining the features that characterize POLQ-mediated deletions, a mutagen, *i*.*e*. UV/TMP, that leaves a signature in the final product, and the sheer size of the collection analysed here, we are now able to establish that replication-associated deletion mutagenesis results from the processing of two opposing 3’ extendable ends, hence a DSB. Above, we have shown that a nascent strand blocked at a site of base damage can serve as a single nucleotide primer to be extended, using a donor sequence, located 50–1,000 bp away, as a template. In Fig 8, we show that there is an equal likelihood of finding the reciprocal event: that the sequence immediately upstream of the blocked fork has served as a template for a priming, reactive end that is located 50–1,000 bp more downstream. This argues that POLQ-mediated repair, as in repairing bona fide DSBs, here acts to connect two 3’ reactive ends. It is currently unknown whether POLQ-mediated repair of replication-associated DSBs necessitates end-resection to create sizable 3’ ssDNA regions (which then function as primer or as template). *In vitro*, human POLQ can extend ssDNA molecules intra-molecularly through a fold-back-stimulated templated reaction [28]. Here, by probing the delins for inserts that had a reverse-complement orientation with respect to their flanking matches we indeed found *in vivo* support for 3’ extension in which both the primer and the template reside on the same DSB end (S7 Fig).

## Discussion

In this study, we have shown that EMS and UV/TMP-induced DSBs are predominantly repaired via POLQ-mediated repair and in-depth analysis of ~7,000 unique deletion footprints allowed us to unveil important characteristics of the *in vivo* repair mechanism. We found that mutagen-induced deletions are the product of alternative DSB repair in which one end is produced by the replication machinery that approached the damage up to one nucleotide. Base pairing of the terminal nucleotide of the blocked nascent strand to single stranded DNA at the opposite break end primes POLQ to polymerize, resulting in DNA tracts that are templated by the sequence immediately flanking the DSB. Further processing of the ensuing stable joints produces simple deletions. However, in case DNA synthesis is interrupted, likely resulting from POLQ errors, a primer-template switch is induced in which the newly formed terminal nucleotides again pair in order for POLQ-mediated extension to continue. We find that one or more cycles of such templated DNA synthesis and primer-template switching can fully explain the composition of deletions that are associated with inserts.

From a conservative point of view, POLQ-mediated repair is a surprisingly elegant solution to the problem how to repair a DSB while keeping the loss of genetic information to an absolute minimum: the repair reaction does not depend on removal of nucleotides to create ligatable ends. It is thus an intriguing idea that nature, perhaps because of the polarity in DNA synthesis being in a 5’ to 3’ direction, has evolved DNA repair and recombination mechanisms that use or tolerate extensive 5’ but not 3’ end-resection; it is obvious that having both these activities prominently used inside nuclei would constitute a great threat to genomes. We have shown here for POLQ-mediated repair of DSBs that the 3’ end of a DNA molecule is very stable and acts as a nucleation site in the repair reaction. Using a specialized polymerase to extend and as such stabilize minimally paired 3’ ends, as opposed to trimming by exonucleases provides a simple yet powerful and versatile solution to a complex problem. One striking aspect of *C*. *elegans* POLQ is the notion of single nucleotide homology. The degree of microhomology in (POLQ-dependent and potentially POLQ-independent) alternative end-joining in a number of other biological systems, such as mouse, human and also plants appear to concern more bases, frequently 3 to 4 bp [4,10,29]. It is yet unclear whether this difference reflects species specific adaptation to the enzyme or differences in the context in which POLQ was studied: a recent *in vitro* study using purified human POLQ demonstrated pairing and extension of 3’ overhangs with just two nucleotides of homology [11]. Another perhaps more striking difference in POLQ-mediated repair between species is the composition of insertions that are found in between the break junctions. While insertions in *C*. *elegans* are mostly derived from a single proximal location, footprints in other species suggest that POLQ is more promiscuous, because inserts often originate from multiple locations, which is suggestive of iterative rounds of abortive repair [4,29]. It is currently unknown what is the cause of this apparent discrepancy between POLQ-mediated repair in different species, but it is of interest to note that mammalian POLQ has evolved to include three additional loop regions in the polymerase domain. One of these loops, loop2, was recently implicated in non-templated terminal transferase activity [28]. The ability to add random nucleotides to the 3’ end of a DSB-repair intermediate may help to generate more opportunity for microhomology-mediated templated resolution.

We have previously shown that POLQ is the primary pathway acting on DSBs that result from DNA replication blocking endogenous lesions [5–7]. An intriguing question concerns the size distribution of resulting deletions: as also shown here, one junction is defined by the replication fork impediment, but what defines the other end? Genetic and molecular dissection of replication-obstructing G-quadruplex structures has led to the model where a replication-stalling DNA lesion results in a ssDNA gap downstream of the impediment [5–7]. More recently, we provided evidence supporting the idea that it is this gap that is responsible for a DSB (with ends 50 to a few hundred bps apart) when the gapped strand is replicated in the next S-phase [7]. POLQ-mediated alternative end-joining subsequently acts on these replication-associated DSBs, instead of HR, which cannot repair the break using the sister chromatid as the latter still contains the replication-blocking impediment (see [7] for details).

In this study, we demonstrate an identical genetic requirement for the repair of DSBs resulting from mutagen exposure; however, it is yet uncertain which replication-blocking lesions are causative. EMS induces a plethora of lesions [30] some of which have been shown to be potent blocks of the replicative polymerases [31], whereas UV/TMP treatment generates psoralen monoadducts on thymines and interstrand crosslinks with a great preference for thymines. Whether deletions induced by UV/TMP are the result of ICL or monoadducts is an outstanding question because the notion of preferential junction formation at 5’TA sites is not discriminatory. Although this outcome perfectly fits a scenario of replication up to the first damaged base of juxtaposed T-T ICLs, it also fits to replication blocking at monoadducts that are preferentially induced at 5’TA sites. The hypersensitivity of *C*. *elegans* POLQ mutant animals towards alkylating and crosslinking agents (as also observed for POLQ/Mus308 mutant *Drosophila*) may seem to contradict to an apparent lack of sensitivity in other systems, such as POLQ knockout mouse cells. We suspect this difference to primarily originate from the fact that *C*. *elegans* toxicity assays, especially those encompassing early embryonic cell divisions, are very sensitive to perturbations of DNA replication [12,32].

Exposure to mutagens, such as EMS and UV/TMP, is widely used to induce random mutations in a great variety of organisms other than *C*. *elegans*, such as *Drosophila*, *Zebrafish*, *Arabidopsis*, Tomato, and mouse. Although EMS-induced damage predominantly induces SNVs, in all these biological systems deletions have been observed ranging in size from a few base pairs to numerous kb [18,20,33–39], and it will be of great interest to investigate whether the causal involvement of POLQ-mediated repair is evolutionary conserved.

In this work, we have linked a specific type of mutations, i.e. deletions of small size, to carcinogenic mutagens that are used in clinical setting. It is becoming increasingly important to establish causal relationships between the exact type and nature of their DNA damaging agents and genome alterations, especially because of the growing interest in mutational signatures in cancer genomes. Recently, the altered genomes of cancer cells are not only inspected for potentially cancer promoting (driver) mutations but also for signatures that testify to the history of the tumour, with respect to genetic makeup and/or environmental exposure [40]. Currently, the majority of these signatures are based on single base substitutions and their surrounding DNA context, but cancer genomes are loaded with copy number variations, deletions and insertions, and also gross chromosomal rearrangements that are likely resulting from mutagenic DNA repair processes [41,42]. It will be interesting to inspect cancer genomes, especially those evolving in cancer cells that are characterized by a defect in homologous recombination for genomic scars that carry the signature of POLQ-mediated end joining, to also determine the contribution of this mutagenic pathway to tumorigenesis.

## Methods

### *C*. *elegans* genetics

Standard methods and conditions for culturing *C*. *elegans* were used [15]. The alleles used in this study were: *polh-1*(lf31); *polq-1* (tm2026); *fcd-2* (tm1298). Bristol N2 was used as wild type in all experiments.

### Nematode mutagenesis

Mutagenesis with EMS was performed at 12.5mM, 25mM, 50mM or 100mM according to standard protocols [15]. In brief, populations were synchronized by alkaline hypochlorite treatment and eggs were allowed to hatch o/n. L1 worms were plated out on 9cm NGM agar plates seeded with *E*. *coli* (OP50) and grown at 20 degrees. Two days later L4 worms were washed off the plates and treated for 4 hours with EMS dissolved in M9.

A similar staging protocol was used for UV/TMP mutagenesis. Subsequently, animals of the L4 stage were treated for one hour with 10μg/ml TMP (Sigma, T6137, stock: 100mg dissolved in 40ml acetone) dissolved in M9. Animals were distributed onto non-seeded NGM plates and exposed to UVA irradiation (366nm; CAMAG 29200 Universal UV LAMP) at a dose rate of 160μW/cm^2^ (Blak-Ray UV-meter model no. J221), after which the animals were transferred to standard OP50/NGM plates.

### Sensitivity assays

Staged animals were exposed to either EMS or UV/TMP at the L4 larval stage and per experimental condition four plates each containing three worms were started. After a 24-36-hour period of egg laying the mothers were removed. The number of (dead) eggs and hatched progeny (after 24 hours) was determined. All experiments were performed in triplicate. We determined the brood size for animals by collecting eggs from individual hermaphrodites in sequential periods of 24 hours. For each period the number of (dead) eggs and hatched progeny (after 24 hours) was determined and then added.

### Deletion library PCR assay

For each deletion library ~80,000 animals were used for synchronization by hypochlorite treatment (0.5M NaOH, 2% hypochlorite) and overnight starvation. Animals of the L4 stage were treated with EMS (50mM), UV/TMP (50 J/m^2^) or mock-treated. P0 animals were removed by hypochlorite treatment 24 hours post-UV/TMP-treatment, and after o/n hatching ~100,000 F1 animals were transferred to 10 9 cm plates and were grown for two days at 20 degrees. Then, animals were collected by rinsing the plates with M9 and distributed over 10 96-well plates such that each well contained ~100 worms in a 5 μl volume. To this 10 μl of lysis buffer was added and animals were subsequently subjected to a standard lysis protocol to liberate the DNA. All 10 plates were pooled into 1 master plate (using 10 μl original DNA mixture), which was used for another round of pooling by combining 10μl from each of the eight wells in a column, finally yielding one row of 12 wells for library. Prior to performing nested PCRs for eight different genomic targets (see S1 Table), the DNA was digested with the thermostable restriction enzyme PspGI. Upon detection of a smaller-than-wild-type product in the pools, PCRs were repeated on the master plate and then on individual plates. The PCR products of the samples that remained positive during this deconvolution exercise (in duplicate) were sequenced. We considered a result a false positive if the samples of lower complexity failed to reproduce the PCR product.

### Bioinformatic analyses

The sequence information for publically available deletion alleles was retrieved from WormBase (WS243). A custom Java program was written to analyse and annotate the WormBase alleles (available upon request). We included a number of additional stringency criteria: 1) the coordinates of the allele should match the information about the allele’s left and right border sequence, 2) insertions within deletions should be as minimal as possible, 3) insertions that contained one or more Ns were discarded. In addition, for cases where sequence homology at the junctions allowed for more than one possible mapping position we placed the homology at the retained flank of the 5’ side. To identify the origin of the insertions in the delins alleles we i) performed BLAST for insertions ≥15 nt, and ii) used a custom-made algorithm aimed to find the longest common substring, i.e. the longest possible match between a stretch of the insertion (≥5 nt) and the sequence that is in close proximity of the junctions (≤50 nt of each flank and 50 nt within either side of the deletion). All deletion alleles used in our analyses can be found in S3 Table.

## Supporting Information

S1 Fig Increased inheritable genetic defects in POLQ-deficient animals exposed to EMS and UV/TMP. **A-B**. The surviving fraction for the broods of P0 animals that were treated with either EMS (A) or UV/TMP (B) was determined. Lines represent the median for each dataset. **C-D**. The surviving fraction was determined for the broods of F1 animals that were born out of P0 animals treated with either EMS (C) or UV/TMP (D). Ten animals were analysed for untreated animals, while 50 treated animals were analysed for each genotype. Lines represent the median for each dataset.
(PDF)

S2 Fig Mutagen-induced deletions require POLQ. **A**. Schematic illustration of how mutagenesis libraries are constructed and screened for deletions at specific loci. For each genotype ~80,000 synchronized P0 L4 animals were mutagenized using EMS or UV/TMP. One day after exposure P0s were removed by hypochlorite treatment and eggs were allowed to hatch o/n in M9. In total ~100,000 F1 animals were used to generate a library in which each well of a 96-well plate contained ~100 animals. DNA of 10 plates was first pooled into one plate and subsequently all columns of this plate were pooled together to create 12 screen samples. We used a strategy based on the use of thermostable restriction enzymes to find deletion alleles [43]. This strategies employs the fact that wild-type template is digested prior to and during PCR amplification, while deletion alleles that lost the recognition site of the restriction enzyme are resistant to this digestion, leading to their preferred amplification. All initial hits were deconvoluted first by PCR of the pooled and then by PCR of the non-pooled samples. Hits that were confirmed (in duplicate) in the non-pooled samples were sequenced. **B-C**. Quantification of the screens for the indicated genotypes and conditions. Fisher’s exact test was used to determine statistical significance: * represents p < 0.05. **D**. The size distribution of deletions that were isolated using the *unc-93* reversion assay; smaller than wild-type bands were sequenced by Sanger sequencing. Each triangle represents a deletion either in *unc-93*, *sup-9* or *sup-10*.
(PDF)

S3 Fig Damage-specific signatures in deletion profiles. **A**. Schematic illustration of the concept that one junction of DNA-damage-induced deletions is defined by the nascent strand blocked at sites of DNA damage. In this hypothesis, the replication-blocking lesion may dictate position -1, being the outermost nucleotide of the lost sequence. **B**. The base composition of all breakpoints, normalized to the relative AT/CG content around the breakpoints (from +100 to -100) for EMS- induced deletion alleles. Position +100 to +1 reflects the sequence that is retained in the deletion alleles; position -1 to -100 reflects the sequence that is lost. Dashed lines represent three times the SD. Data points outside these boundaries are marked with a dot. **C**. As in B, but only for delins. **D**. The tandem base composition of all breakpoints, normalized to the relative di-nucleotide occurrence at position +200 to -200. For each indicated position (+ for retained sequence;—for lost sequence) the base composition is coupled to the composition of the immediate downstream base. Only dinucleotides that were found elevated (>3.5 times the SD) are depicted in the legend, with elevated data points marked with a dot. Only the largest and smallest variations for individual dinucleotides are shown. **E**. As in D, but only for UV/TMP-induced delins.
(PDF)

S4 Fig Deletions result from a single replication-blocking lesion. **A**. A Venn-diagram representation of delins (*n* = 1,588) in which the blue and red circles indicate the number of delins that have a 5’TA at position +1,-1 at the 5’ or 3’ side of the deletion (see Figs 3 and 5). The overlapping area represents the number of cases where a 5’TA was found at both sides of the deletion. UV/TMP-induced deletions are thus characterized by a single 5’TA at only one breakpoint. **B**. A histogram depicting the observed and the expected number of delins that are flanked by 5’TA. The expected number is calculated for two scenarios: i) the probability of finding a second 5’TA is equally overrepresented as finding a 5’TA at a given breakpoint (which would argue that a delins results from a crosslink at both breakpoints), or ii) the probability of finding a second 5’TA is equal to its probability for any given sequence (which would argue that only one crosslink underlies the genesis of a delins).
(PDF)

S5 Fig The origin of insertions in EMS and UV/TMP-induced delins. **A**. Distribution of insertions that originate from inter- (>1,000 bp away from deletion) or intrachromosomal locations relative to the position of the delins. **B.** The distance between the positions of the delins and the location from where the templates originate is plotted (in bp) for delins with intrachromosomal inserts that do not map to the immediate vicinity.
(PDF)

S6 Fig POLQ signature is diminished in large deletions. Distribution of all deletion alleles binned to size. For each bin the categories delins, no homology, 1 bp of homology and >1 bp homology are shown. The number of events in each bin is shown in the upper panel.
(PDF)

S7 Fig Snap-back replication represents a minor part of delins. **A)** Schematic illustration of the concept that templated insertions can result from extending the 3’ hydroxyl end of a DSB end using i) its flanking sequence in-cis through snapback interaction (left drawing), or ii) the other end of the DSB in trans (right drawing). Note that snap-back replication results in insertions that are of reverse-complement configuration with respect to the sequence in the flank that served as a template. **B**. Visual representation of the origins of flank insertions in both forward (blue) and reverse-complement (red) orientation. A single line represents one mapped flank insertion and is drawn relative to its cognate breakpoint with ‘-‘ for deleted and ‘+‘ for retained sequences. Only inserts where a significant part of the insert could be traced back (likelihood of finding a longest common substring of that particular size: p < 0.05) were represented.
(PDF)

S1 Table Primers used for deletion detection in EMS and UV/TMP mutagenized libraries. (XLSX)

S2 Table Deletion size annotation discrepancy in alleles created at the National Bioresource Project Japan. (XLSX)

S3 Table Final deletion and delins allele table. (XLSX)
