# Supplementary figures and images for: Genomic Scars Generated by Polymerase Theta Reveal the Versatile Mechanism of Alternative End-Joining
Source: PLoS Genet. 2016 Oct 18;12(10):e1006368. doi: 10.1371/journal.pgen.1006368 (PMC5068794; doi:10.1371/journal.pgen.1006368)

A

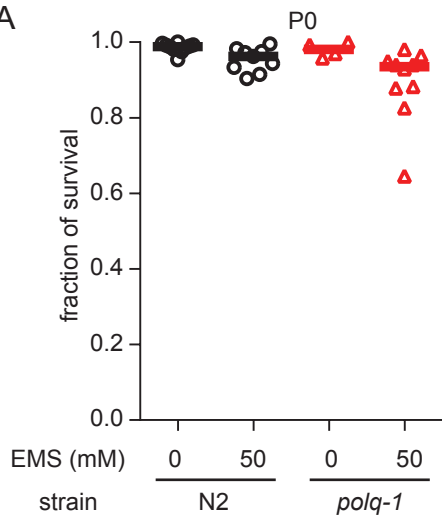

B

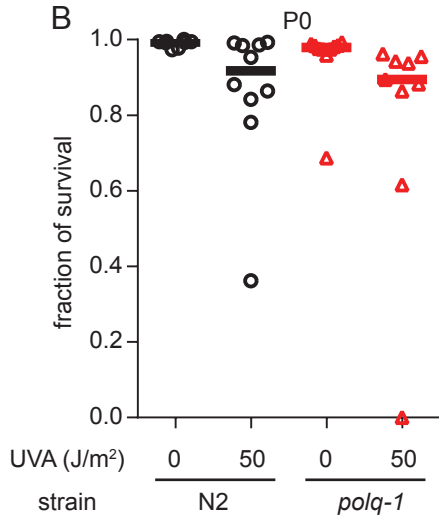

C

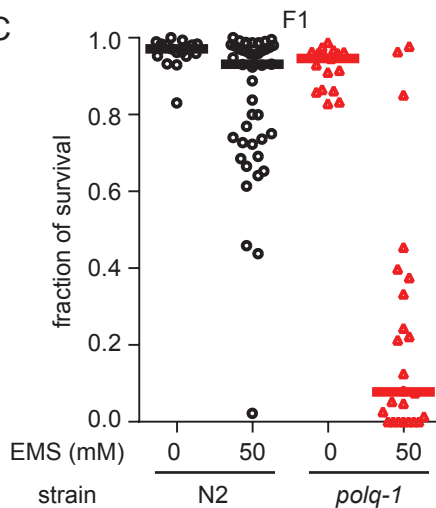

D

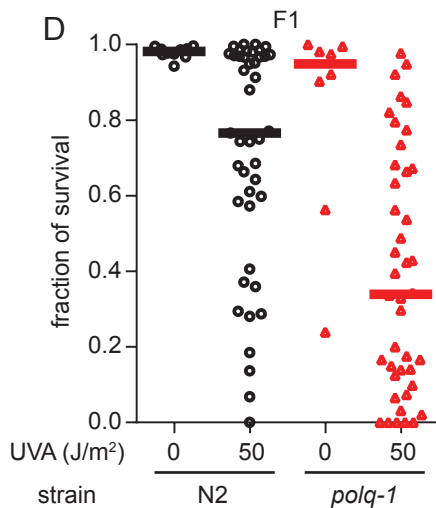

Supplement: S1 Fig — A-B. The surviving fraction for the broods of P0 animals that were treated with either EMS (A) or UV/TMP (B) was determined. Lines represent the median for each dataset. C-D. The surviving fraction was determined for the broods of F1 animals that were born out of P0 animals treated with either EMS (C) or UV/TMP (D). Ten animals were analysed for untreated animals, while 50 treated animals were analysed for each genotype. Lines represent the median for each dataset. (PDF) [file pgen.1006368.s001.pdf]

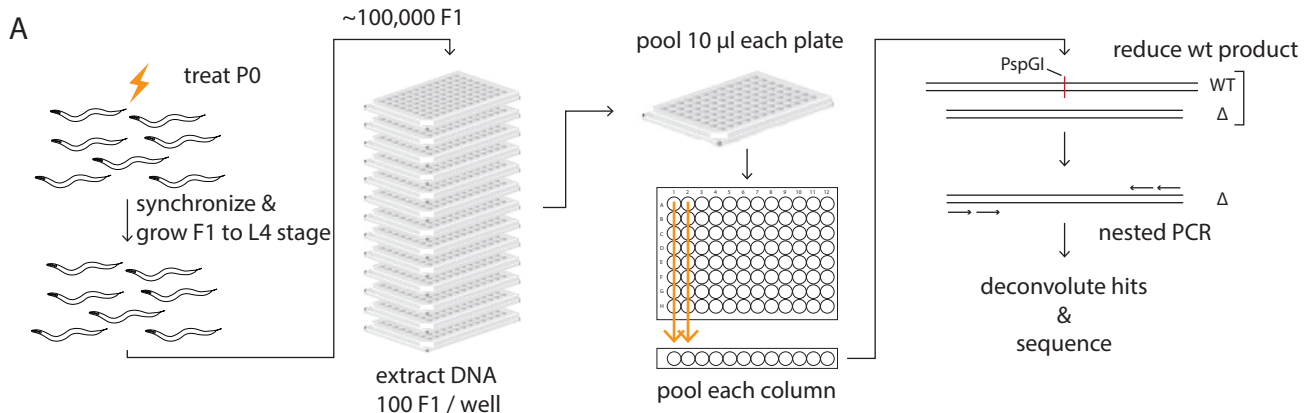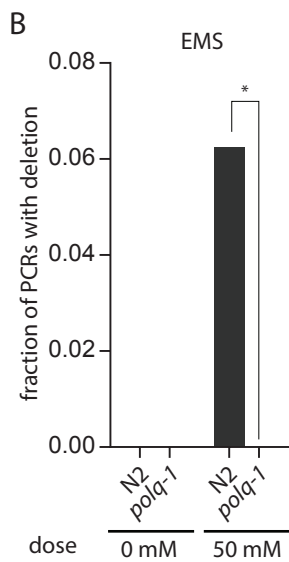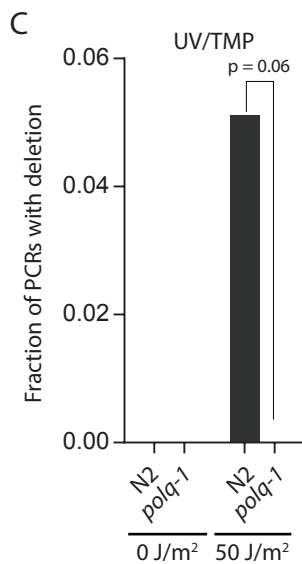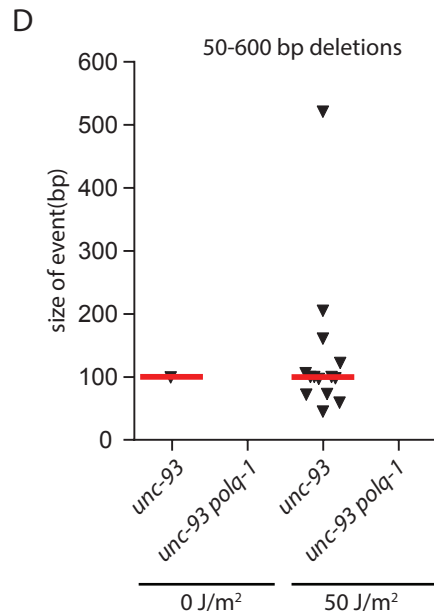

Supplement: S2 Fig — A. Schematic illustration of how mutagenesis libraries are constructed and screened for deletions at specific loci. For each genotype ~80,000 synchronized P0 L4 animals were mutagenized using EMS or UV/TMP. One day after exposure P0s were removed by hypochlorite treatment and eggs were allowed to hatch o/n in M9. In total ~100,000 F1 animals were used to generate a library in which each well of a 96-well plate contained ~100 animals. DNA of 10 plates was first pooled into one plate and subsequently all columns of this plate were pooled together to create 12 screen samples. We used a strategy based on the use of thermostable restriction enzymes to find deletion alleles [43]. This strategies employs the fact that wild-type template is digested prior to and during PCR amplification, while deletion alleles that lost the recognition site of the restriction enzyme are resistant to this digestion, leading to their preferred amplification. All initial hits were deconvoluted first by PCR of the pooled and then by PCR of the non-pooled samples. Hits that were confirmed (in duplicate) in the non-pooled samples were sequenced. B-C. Quantification of the screens for the indicated genotypes and conditions. Fisher’s exact test was used to determine statistical significance: * represents p < 0.05. D. The size distribution of deletions that were isolated using the unc-93 reversion assay; smaller than wild-type bands were sequenced by Sanger sequencing. Each triangle represents a deletion either in unc-93, sup-9 or sup-10. (PDF) [file pgen.1006368.s002.pdf]

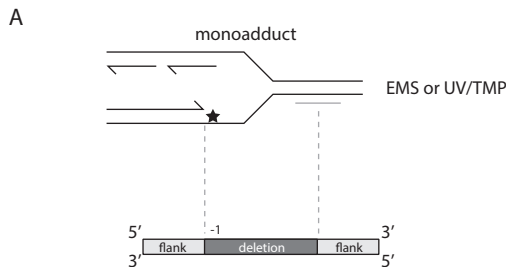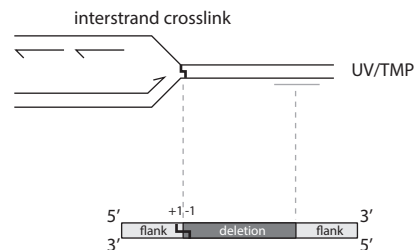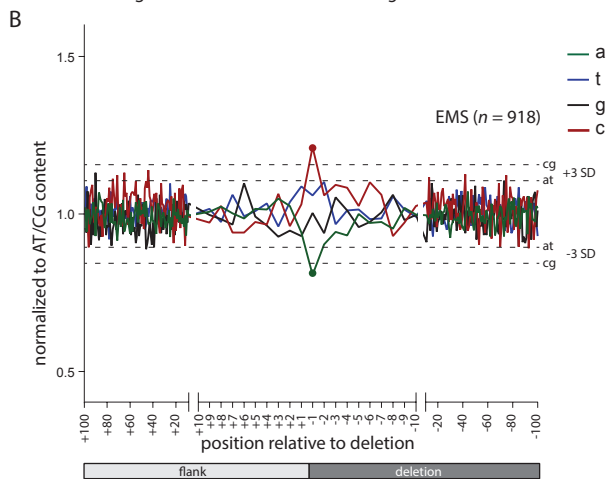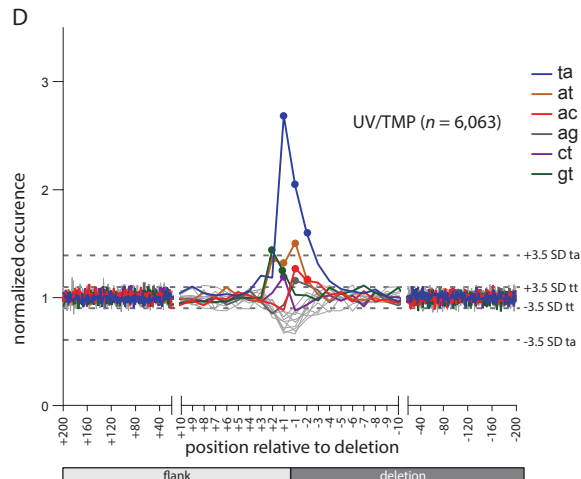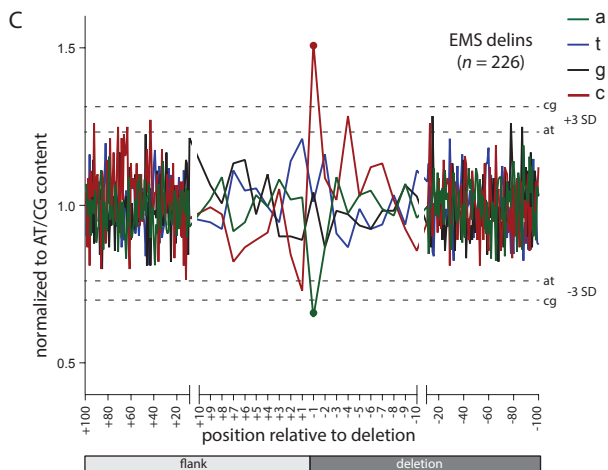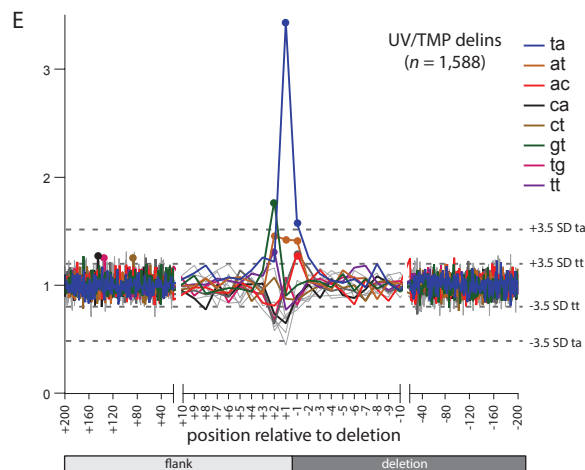

Supplement: S3 Fig — A. Schematic illustration of the concept that one junction of DNA-damage-induced deletions is defined by the nascent strand blocked at sites of DNA damage. In this hypothesis, the replication-blocking lesion may dictate position -1, being the outermost nucleotide of the lost sequence. B. The base composition of all breakpoints, normalized to the relative AT/CG content around the breakpoints (from +100 to -100) for EMS- induced deletion alleles. Position +100 to +1 reflects the sequence that is retained in the deletion alleles; position -1 to -100 reflects the sequence that is lost. Dashed lines represent three times the SD. Data points outside these boundaries are marked with a dot. C. As in B, but only for delins. D. The tandem base composition of all breakpoints, normalized to the relative di-nucleotide occurrence at position +200 to -200. For each indicated position (+ for retained sequence;—for lost sequence) the base composition is coupled to the composition of the immediate downstream base. Only dinucleotides that were found elevated (>3.5 times the SD) are depicted in the legend, with elevated data points marked with a dot. Only the largest and smallest variations for individual dinucleotides are shown. E. As in D, but only for UV/TMP-induced delins. (PDF) [file pgen.1006368.s003.pdf]

A.

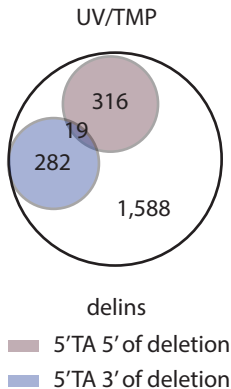

B.

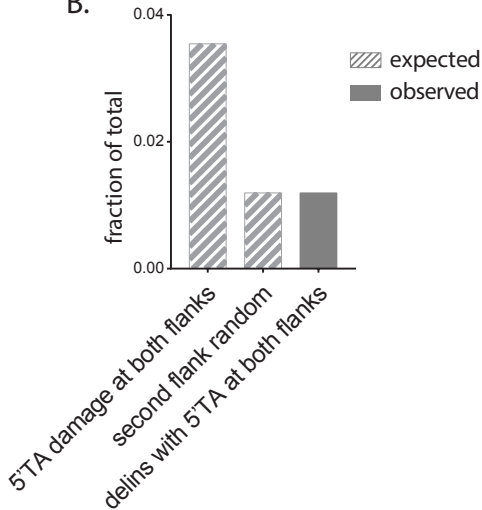

Supplement: S4 Fig — A. A Venn-diagram representation of delins (n = 1,588) in which the blue and red circles indicate the number of delins that have a 5’TA at position +1,-1 at the 5’ or 3’ side of the deletion (see Figs 3 and 5). The overlapping area represents the number of cases where a 5’TA was found at both sides of the deletion. UV/TMP-induced deletions are thus characterized by a single 5’TA at only one breakpoint. B. A histogram depicting the observed and the expected number of delins that are flanked by 5’TA. The expected number is calculated for two scenarios: i) the probability of finding a second 5’TA is equally overrepresented as finding a 5’TA at a given breakpoint (which would argue that a delins results from a crosslink at both breakpoints), or ii) the probability of finding a second 5’TA is equal to its probability for any given sequence (which would argue that only one crosslink underlies the genesis of a delins). (PDF) [file pgen.1006368.s004.pdf]

A

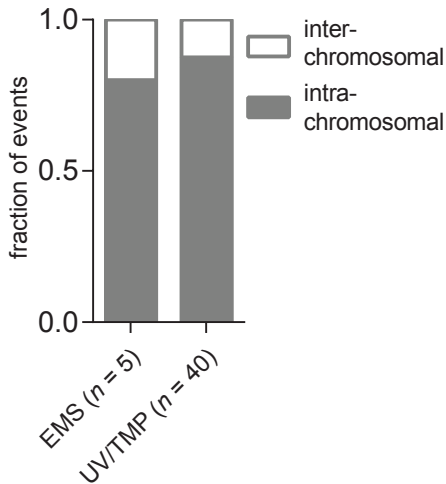

B

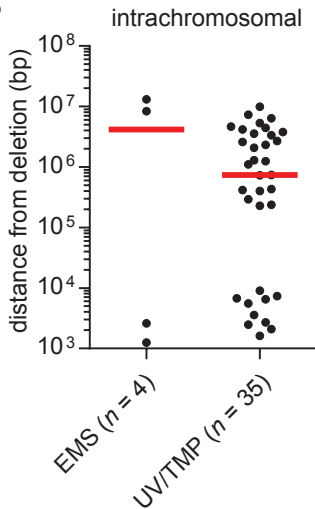

Supplement: S5 Fig — A. Distribution of insertions that originate from inter- (>1,000 bp away from deletion) or intrachromosomal locations relative to the position of the delins. B. The distance between the positions of the delins and the location from where the templates originate is plotted (in bp) for delins with intrachromosomal inserts that do not map to the immediate vicinity. (PDF) [file pgen.1006368.s005.pdf]

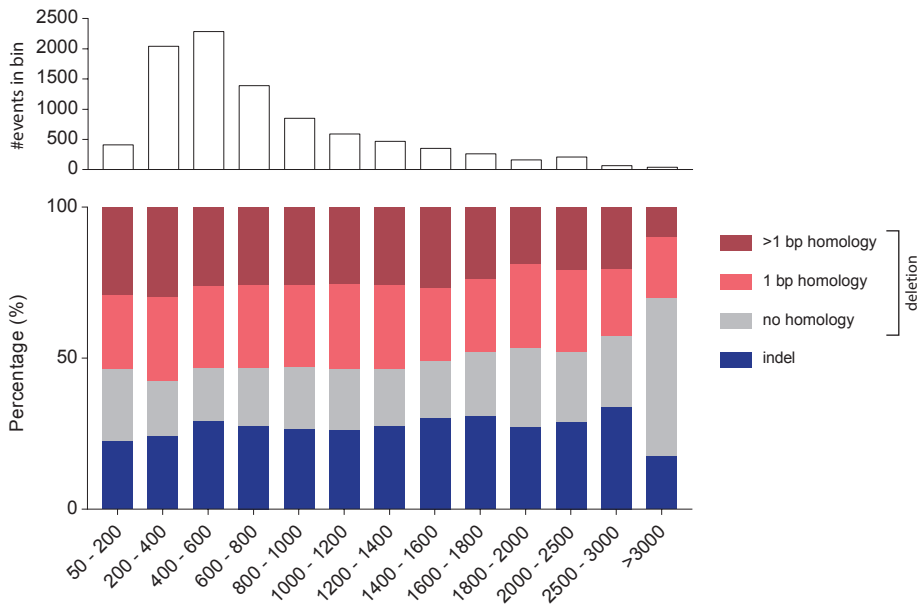

Supplement: S6 Fig — Distribution of all deletion alleles binned to size. For each bin the categories delins, no homology, 1 bp of homology and >1 bp homology are shown. The number of events in each bin is shown in the upper panel. (PDF) [file pgen.1006368.s006.pdf]

A

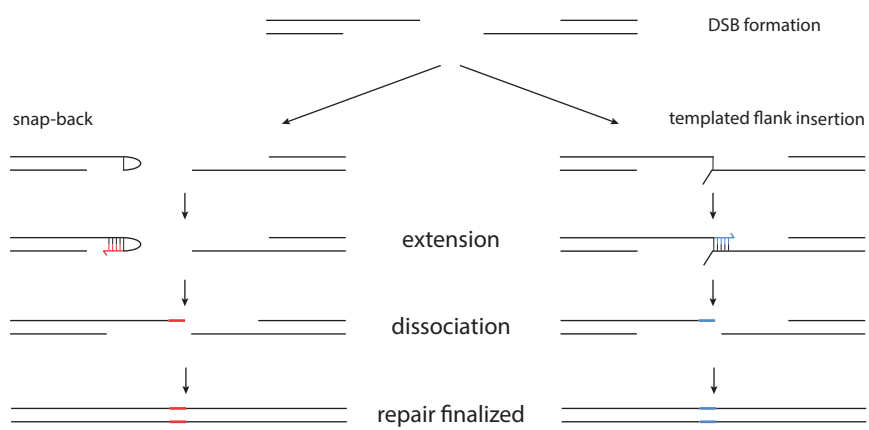

B

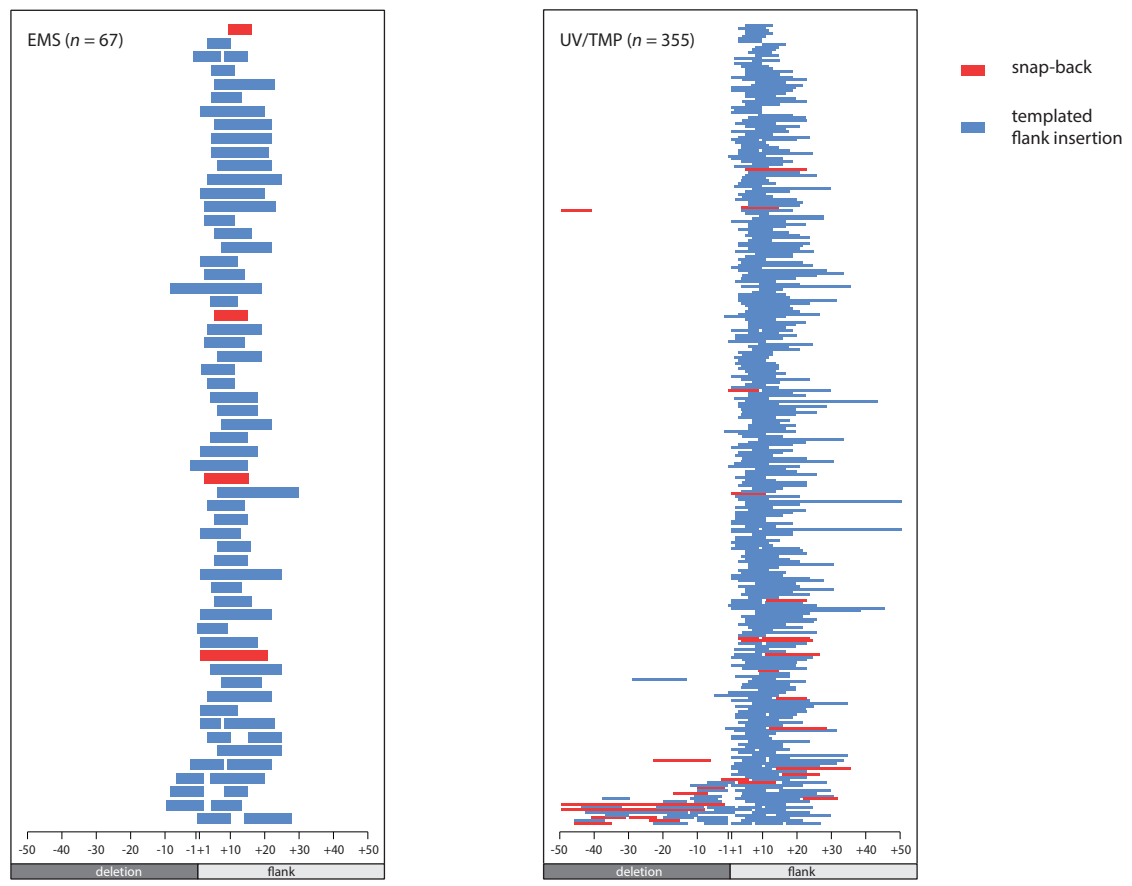

Supplement: S7 Fig — A) Schematic illustration of the concept that templated insertions can result from extending the 3’ hydroxyl end of a DSB end using i) its flanking sequence in-cis through snapback interaction (left drawing), or ii) the other end of the DSB in trans (right drawing). Note that snap-back replication results in insertions that are of reverse-complement configuration with respect to the sequence in the flank that served as a template. B. Visual representation of the origins of flank insertions in both forward (blue) and reverse-complement (red) orientation. A single line represents one mapped flank insertion and is drawn relative to its cognate breakpoint with ‘-‘ for deleted and ‘+‘ for retained sequences. Only inserts where a significant part of the insert could be traced back (likelihood of finding a longest common substring of that particular size: p < 0.05) were represented. (PDF) [file pgen.1006368.s007.pdf]
